# Septic Pylephlebitis and Cavernous Transformation Presenting as Pyrexia of Unknown Origin: A Diagnostic Challenge

**DOI:** 10.1002/ccr3.73382

**Published:** 2026-08-22

**Authors:** Aditi Sarker, Prodipta Chowdhury, Md. Razibul Alam

**Affiliations:** ^1^ Department of Gastroenterology Bangabandhu Sheikh Mujib Medical University Dhaka Bangladesh; ^2^ Department of Endocrinology Bangabandhu Sheikh Mujib Medical University Dhaka Bangladesh

**Keywords:** cholangitis, fever of unknown origin, portal biliopathy, portal hypertension, portal vein thrombosis, pylephlebitis

## Abstract

Acute cholangitis classically presents with fever, jaundice, and right upper quadrant pain, but atypical or subacute disease may present as pyrexia of unknown origin (PUO) and remain unrecognized until vascular complications develop. We report a 45‐year‐old man referred for weight loss, malaise, and suspected non‐cirrhotic portal hypertension after upper gastrointestinal endoscopy showed esophageal varices despite a normal FibroScan. Detailed history revealed intermittent fever for 2 months. Laboratory evaluation demonstrated marked systemic inflammation, with leukocytosis, neutrophilia, ferritin 1095 ng/mL, and C‐reactive protein > 200 mg/L, together with a cholestatic‐hepatitic liver enzyme pattern and preserved liver synthetic function. Blood and urine cultures were negative. Initial ultrasonography showed bile duct dilatation without a definite obstructing lesion, delaying diagnosis. Subsequent magnetic resonance cholangiopancreatography and contrast‐enhanced computed tomography demonstrated biliary abnormality with portal vein thrombosis and periportal collateralization, consistent with septic pylephlebitis, cavernous transformation, and portal biliopathy secondary to occult cholangitis. The patient was treated with intravenous broad‐spectrum antibiotics and anticoagulation, with clinical improvement and partial portal vein recanalisation on follow‐up ultrasonography at 3 months. This case highlights that occult cholangitis should be considered in patients with PUO and cholestatic liver biochemistry, even in the absence of classic biliary symptoms, and that early cross‐sectional biliary imaging may allow timely recognition of pylephlebitis before chronic portal hypertensive and biliary sequelae become established.

## Key Clinical Message

Cholangitis may present as pyrexia of unknown origin (PUO). Advanced imaging is essential for patients with fever and cholestatic liver function tests to detect occult biliary infections. Early recognition prevents septic portal vein thrombosis and subsequent complications such as chronic portal hypertension and portal biliopathy, ensuring timely and effective management.

## Graphical abstract

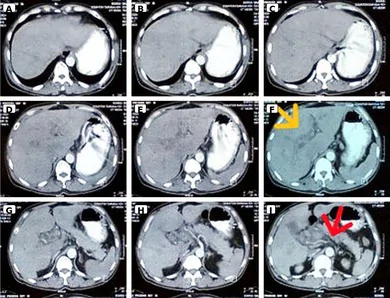

**Abbreviations**
CECT: contrast‐enhanced computed tomography
MRCP: magnetic resonance cholangiopancreatography
PUO: pyrexia of unknown origin
SS‐B/La: Sjögren syndrome type B antibody

## 1 Introduction

Pyrexia of unknown origin (PUO) remains a diagnostic challenge with a broad differential that includes infection, malignancy, non‐infectious inflammatory disease, and miscellaneous causes. In many adults, the final diagnosis depends on a stepwise, clue‐driven approach, with repeated clinical assessment and selective use of advanced imaging rather than depending on the initial presentation alone [1]. Acute cholangitis is a significant and treatable infection, yet the classical Charcot triad of fever, jaundice, and right upper quadrant pain has limited sensitivity [2]. When cholangitis presents without localizing biliary symptoms, diagnosis may be delayed, and complications such as septic portal vein thrombosis (pylephlebitis) can occur [3, 4]. Biliary‐source pylephlebitis is rare, and progression to cavernous transformation with portal biliopathy makes the presentation even more unusual [3, 4, 5, 6, 7].

We report a middle‐aged man who presented with prolonged fever, weight loss, portal hypertensive changes, and preserved liver stiffness, in whom advanced imaging ultimately revealed occult cholangitis complicated by portal vein thrombosis and cavernous transformation.

## 2 Case Presentation and Clinical Examination

A 45‐year‐old male farmer, non‐diabetic and non‐smoker, was referred with unintentional weight loss and malaise. On initial query, he did not volunteer focal biliary symptoms, but he reported a history of irregular fever over the preceding two months. On admission, he was febrile at 37.8°C (100°F), while the remaining vital signs were stable. The patient was not icteric, had no organomegaly, and had no lymphadenopathy.

Before referral, he had been treated empirically for presumed enteric fever and later put on prednisolone 1 mg/kg/day without a steroid‐sparing agent for suspected adult‐onset Still's disease. The fever transiently subsided, but he again became febrile within 02 weeks, so prednisolone was discontinued. His overall condition and weight loss did not improve.

## 3 Differential Diagnosis, Investigations and Diagnosis

At presentation, the differential diagnosis included occult hepatobiliary infection, autoimmune or inflammatory disease, malignancy, and non‐cirrhotic portal hypertension.

Laboratory and ancillary investigations are summarized in Table 1. The patient had marked inflammatory activity with leukocytosis (21.7 × 10^9^/L), neutrophilia (85%), ferritin 1095 ng/mL, and C‐reactive protein > 200 mg/L. Liver biochemistry showed mild‐to‐moderate cholestatic‐hepatitic abnormalities: alkaline phosphatase 251 U/L, alanine aminotransferase 112 U/L, and aspartate aminotransferase 107 U/L. Blood and urine cultures were negative, viral and tumor markers were negative, and thrombophilia screening was unrevealing. The extractable nuclear antigen profile showed isolated anti‐Sjögren syndrome type B (SS‐B/La) positivity, which was not considered sufficient to explain the overall presentation.

**TABLE 1:** Investigation profile.

Parameter	Value	Reference range
Total white blood cell count	**21.7 × 10** ^**9**^ **/L**	4–11 **×** 10^9^/L
Neutrophils	**85%**	40%–80%
Serum ferritin	**1095 ng/mL**	30–400 ng/mL
C‐reactive protein	**> 200 mg/L**	< 10 mg/L
Lactate dehydrogenase	**680 U/L**	135–225 U/L
Alanine aminotransferase	**112 U/L**	10–49 U/L
Aspartate aminotransferase	**107 U/L**	8–48 U/L
Alkaline phosphatase	**251 U/L**	30–130 U/L
Thyroid‐stimulating hormone	2.5 μU/mL	0.5–5 μU/mL
Urea and electrolytes	Normal	Not applicable
Random blood sugar	5.5 mmol/L	< 7.8 mmol/L
Blood culture and sensitivity	No growth	Not applicable
Urine culture and sensitivity	No growth	Not applicable
Mantoux test	2 mm	< 5 mm
Viral markers (HBV, HCV, HIV 1, and 2)	Negative	Not applicable
ICT for malaria and kala‐azar	Negative	Not applicable
Extractable nuclear antigen profile	**Positive (SS‐B/La)**	Negative
D‐dimer	0.5 μg/mL	Up to 0.5 μg/mL
Fibrin degradation products	6 μg/mL	< 10 μg/mL
Protein C and S	Negative	Not applicable
Antithrombin III	Negative	Not applicable
Tumor markers (AFP, CA19‐9, PSA, and CEA)	Negative	Not applicable
Electrocardiogram	Normal	Not applicable
Echocardiography	Normal study	Not applicable
Chest X‐ray (posteroanterior view)	Normal	Not applicable
Colonoscopy	Normal	Not applicable
Transient elastography (FibroScan)	4.5 kPa	2–7 kPa
Duplex study of hepatobiliary system	**Cavernous transformation of the portal vein with mild portal hypertension**	Not applicable

*Note:* The bold values indicate the abnormal ones.

Abbreviations: AFP, alpha‐fetoprotein; CA19‐9, carbohydrate antigen 19–9; CEA, carcinoembryonic antigen; HBV, hepatitis B virus; HCV, hepatitis C virus; ICT, immunochromatographic test; PSA, prostate‐specific antigen; SS‐B/La, Sjögren syndrome type B antibody.

**FIGURE 1:**
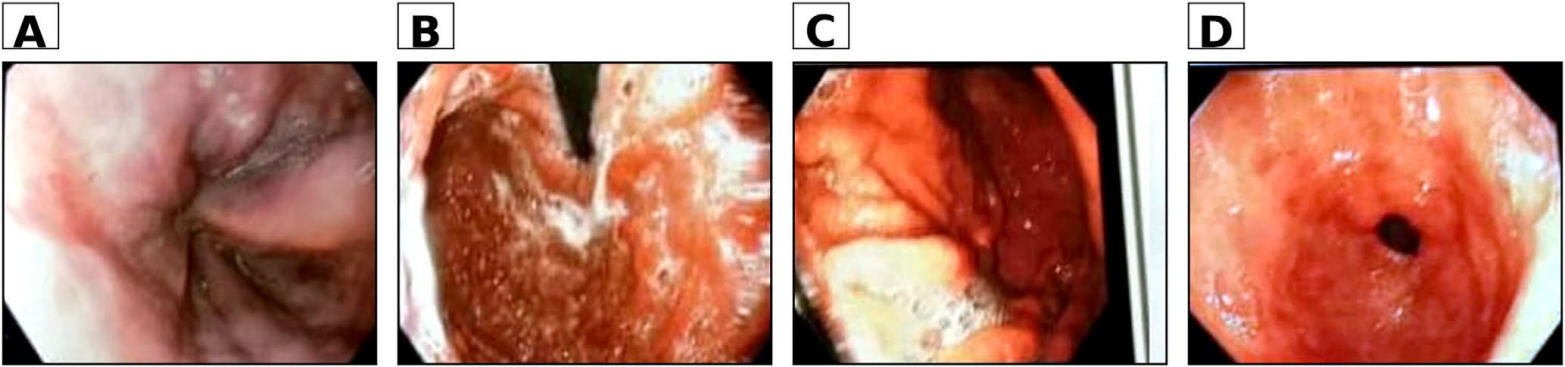
Upper gastrointestinal endoscopy. Panels A and B demonstrate Grade II–III esophageal varices in the distal esophagus. Panels C and D show portal hypertensive gastropathy with a mosaic mucosal pattern.

Upper gastrointestinal endoscopy revealed Grade II‐III esophageal varices and portal hypertensive gastropathy (Figure 1), whereas transient elastography was normal (FibroScan 4.5 kPa), making cirrhosis less likely.

**FIGURE 2:**
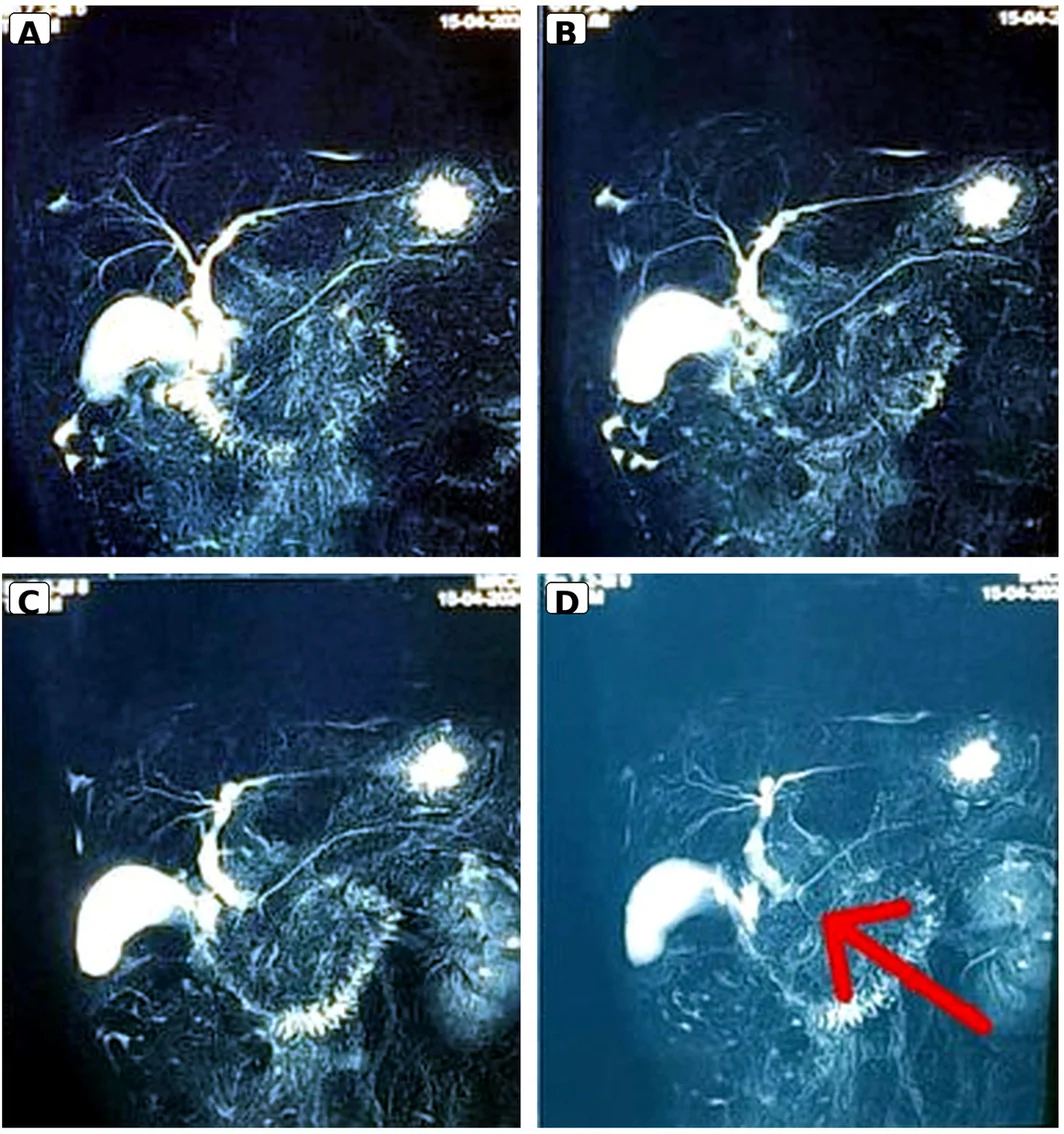
Magnetic resonance cholangiopancreatography (MRCP). Panels A–D show abnormal hepatobiliary imaging with features of portal vein thrombosis and cavernous transformation (portal cavernoma). Panel D highlights the abnormality with an arrow.

**FIGURE 3:**
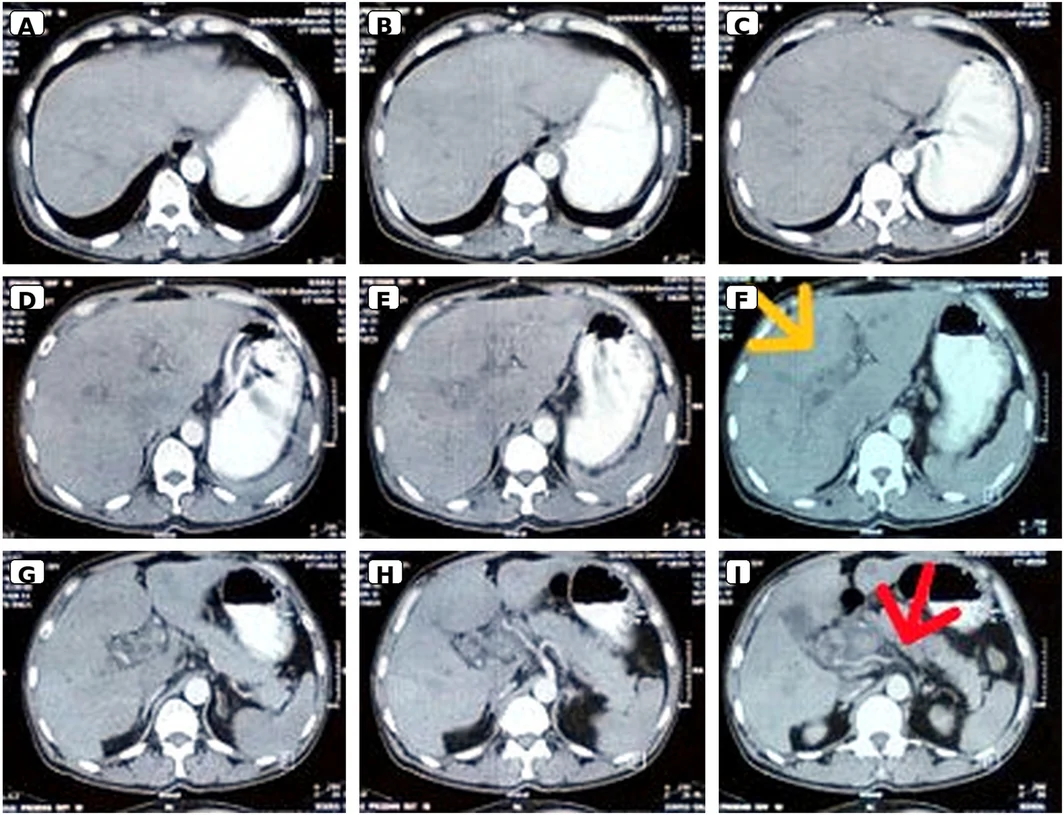
Contrast‐enhanced computed tomography (CT) of the abdomen. Panels A–I demonstrate portal vein thrombosis with periportal serpiginous collateral vessels, consistent with cavernous transformation. Red arrowhead: Thrombus in portal vein, Yellow arrowhead: Dilatation of CBD and intrahepatic biliary tree.

Earlier ultrasonography, performed during the febrile phase, demonstrated bile duct dilatation without an obvious obstructing stone or definite stricture, which delayed identification of the source. Magnetic resonance cholangiopancreatography (MRCP) subsequently demonstrated abnormal biliary features together with portal vein thrombosis and collateralisation suggestive of cavernous transformation (Figure 2). Contrast‐enhanced computed tomography (CECT) of the abdomen then confirmed portal vein thrombosis with periportal serpiginous collateral vessels, establishing chronic portal vein thrombosis with cavernous transformation and portal biliopathy secondary to occult cholangitis (Figure 3).

## 4 Treatment

The patient received intravenous broad‐spectrum antibiotics (Ceftriaxone 2 g twice daily for 02 weeks) and supportive care, with defervescence and improvement in inflammatory markers. Therapeutic anticoagulation (rivaroxaban 10 mg once daily for 03 months) was initiated after endoscopic documentation of Grade II‐III esophageal varices and individual bleeding‐risk assessment. Because cavernous transformation coexisted with biliary dilatation and portal hypertensive changes, portal cavernoma cholangiopathy (portal biliopathy) was considered, and the patient was managed conservatively with portal hypertension prophylaxis and multidisciplinary follow‐up.

## 5 Outcome and Follow‐Up

At 3 months, the patient reported no fever, marked symptomatic improvement, and good appetite. Physical examination was unremarkable with 2 kg weight gain. Follow‐up duplex ultrasonography demonstrated partial recanalization of the portal vein.

## 6 Discussion

PUO, defined as fever exceeding 38.3°C (101°F) for over three weeks without an identifiable cause despite thorough evaluation, posed a significant diagnostic challenge [1]. In this patient, the combination of prolonged fever, weight loss, negative basic microbiology, and preserved liver stiffness initially obscured the diagnosis. However, marked leukocytosis, neutrophilia, and very high C‐reactive protein supported a significant systemic inflammatory process, while the concurrent elevation of alkaline phosphatase with mild–moderate aminotransferase rise suggested a cholestatic‐hepatitic pattern, thereby increasing suspicion for an occult hepatobiliary source. This constellation is consistent with the Tokyo Guidelines framework, in which systemic inflammation together with abnormal liver biochemistry should prompt targeted biliary imaging even when classic localizing symptoms are absent [2].

Although acute cholangitis is classically associated with Charcot's triad, many patients do not present with all three components, and atypical or subacute disease may manifest chiefly as fever [2]. This explains why initial ultrasonography showing only non‐specific biliary dilatation was insufficient. In such cases, MRCP and CECT may reveal occult biliary infection and its vascular consequences [3, 4]. Pylephlebitis secondary to cholangitis appears to be distinctly uncommon; published biliary‐source cases are largely limited to isolated reports, including those of Ozawa and Shikino and Zardi et al. [3, 8]. This is consistent with larger reviews in which diverticulitis and appendicitis, rather than cholangitis, predominate as the infective source of pylephlebitis [4, 5].

Pylephlebitis is thought to arise from local infectious spread to the portal venous system, endothelial injury, and a systemic prothrombotic inflammatory state [4, 5]. Once portal vein thrombosis becomes chronic, cavernous transformation and portal hypertension may follow, leading to portal cavernoma cholangiopathy from extrinsic compression and ischaemic injury to the bile ducts [6, 7]. Current reviews support anticoagulation for at least 3–6 months in acute or recent portal vein thrombosis when bleeding risk is acceptable, together with treatment of the underlying cause [6]. Our patient's favorable short‐term course and partial recanalisation on follow‐up support the value of timely recognition, antibiotics, anticoagulation, and multidisciplinary surveillance.

A limitation of this report is the absence of microbiological confirmation of the biliary source. Nevertheless, the inflammatory profile, cholestatic liver test abnormalities, biliary imaging findings, and subsequent clinical response together make occult cholangitis the most plausible explanation. Overall, this case highlights that occult cholangitis should remain in the differential diagnosis of PUO when fever coexists with cholestatic liver biochemistry, even in the absence of abdominal pain or jaundice, and that early advanced biliary imaging may reduce the risk of chronic portal hypertensive and biliary sequelae [2, 3, 4, 5, 6, 7].

## Author Contributions

**Aditi Sarker:** conceptualization, data curation, investigation, writing – original draft. **Prodipta Chowdhury:** formal analysis, methodology, resources, software, visualization, writing – review and editing. **Md. Razibul Alam:** conceptualization, investigation, methodology, resources, supervision, validation, writing – review and editing.

## Funding

The authors have nothing to report.

## Ethics Statement

This case report was prepared in accordance with accepted ethical standards for anonymised clinical reporting.

## Consent

Written informed consent was obtained from the patient for publication of this case report and the accompanying images.

## Conflicts of Interest

The authors declare no conflicts of interest.

## Data Availability

The data supporting the findings of this report are available from the corresponding author upon reasonable request and will be made available to the journal for review if required.
